# How to build personalized multi-omics comorbidity profiles

**DOI:** 10.3389/fcell.2015.00028

**Published:** 2015-06-24

**Authors:** Mohammad Ali Moni, Pietro Liò

**Affiliations:** ^1^Computer Laboratory, University of CambridgeCambridge, UK; ^2^Department of Computer Science and Engineering, Pabna University of Science and TechnologyPabna, Bangladesh; ^3^Bone Biology, Garvan Institute of Medical Research, The University of New South WalesSydney, NSW, Australia

**Keywords:** comorbidity, multi-omics, ontology, multiplex network, data integration

## Abstract

Multiple diseases (acute or chronic events) occur together in a patient, which refers to the disease comorbidities, because of the multi ways associations among diseases. Due to shared genetic, molecular, environmental, and lifestyle-based risk factors, many diseases are comorbid in the same patient. Methods for integrating multiple types of omics data play an important role to identify integrative biomarkers for stratification of patients into groups with different clinical outcomes. Moreover, integrated omics and clinical information may potentially improve prediction accuracy of disease comorbidities. However, there is a lack of effective and efficient bioinformatics and statistical software for true integrative data analysis. With the availability of the wide spread huge omics, phenotype and ontology information, it is becoming more and more practical to help doctors in clinical diagnostics and comorbidity prediction by providing appropriate software tool. We developed an R software POGO to compute novel estimators of the disease comorbidity risks and patient stratification. Starting from an initial diagnosis, omics and clinical data of a patient the software identifies the association risk of disease comorbidities. The input of this software is the initial diagnosis of a patient and the output provides evidence of disease comorbidities. The functions of POGO offer flexibility for diagnostic applications to predict disease comorbidities, and can be easily integrated to high–throughput and clinical data analysis pipelines. POGO is compliant with the Bioconductor standard and it is freely available at www.cl.cam.ac.uk/~mam211/POGO/.

## Introduction

Exploring disease-disease associations by using multi-omics and clinical information is expected to improve our current knowledge of disease relationships, which may lead to further improvements in disease diagnosis, prognosis and treatment (Park et al., 2009). Recent research has increasingly demonstrated that many seemingly dissimilar diseases have common molecular mechanisms and strong associations among them (Yu et al., 2015). Because of the associations among diseases, multiple diseases (acute or chronic events) occur together in a patient, which is called disease comorbidities. Comorbidities relationships exist among diseases whenever they impact the same patients significantly more than expected by chance (Žitnik et al., 2013). It represents the co–occurrence of diseases or presence of different illness or medical conditions simultaneously or one after another in the same patient (Hidalgo et al., 2009; Park et al., 2009). The set of sequential disease associations, which refers to disease trajectories, uncovers time based disease comorbidity associations. They can also form the basis for understanding mathematical properties of co-morbidity networks (Hidalgo et al., 2009; Jensen et al., 2014). Comorbidity associations can be due to direct or indirect causal relationships and the shared risk factors among them (Tong and Stevenson, 2007). If two diseases have comorbidity association, the incidence of one of them in an individual may increase the likelihood of another disease occurring. Certain diseases, such as diabetes and obesity often co-occur in the same patient, sometimes one being considered a significant risk factor for the other (Lee et al., 2008). Disease comorbidities are increasingly placing a greater burden on individuals, societies and health care services. It is an important factor for better risk stratification of patients and treatment planning.

Diseases with similar molecular, environmental, and lifestyle risk factors may be comorbid in individuals or may be risk factors for another disorder (Davis et al., 2010). Shared genetic, environmental and lifestyle factors have similar consequences, increasing the co-occurrence of associated diseases in the same individual. So, a person diagnosed for a combination of disorders and exposed to particular environmental, lifestyle and genetic risk factors may be at a increased risk of developing several other genetically and environmentally associated diseases (Barabási et al., 2011). It is now well accepted that phenotypes are determined by genetic material under environmental influences. For instance, many well-known and influential lifestyle factors such as smoking, diet, and alcohol intake are actively related to diabetes type 1 and type 2, and obesity (Astrup, 2001). Moreover, many complex diseases, such as cancer and diabetes, are affected by an integrated effect of environment and epistasis among many genes (Davis et al., 2010).

Recent evidence has exhibited that microRNAs play key roles in the evolution and progression of human diseases. Functionally related microRNAs tend to be associated with phenotypically similar diseases (Lu et al., 2008). Recently, genome-wide association studies (gwas) proved to be useful as a method for exploring phenotypic associations with diseases (Lewis et al., 2011). Single-nucleotide polymorphisms (SNPs), a variation of a single nucleotide, are assumed to play a major role in causing phenotypic differences between individuals. It has become possible to assess systematically the contribution of common SNPs to complex diseases. Copy number variations (CNVs; which involve loss, duplication or rearrangement of long stretches of DNA in individual's genome) can cause various phenotypic abnormalities (Zhang et al., 2009). CNVs are significantly associated with the risk of complex human diseases including inflammatory autoimmune disorders, diabetes etc. (Bae et al., 2011). The development of type 2 diabetes has also been known to be influenced by molecular, lifestyle and environmental factors (Kahn et al., 2006).

Most of the research works focussed on a particular data type, for example gene expression, to find profiles that are associated with particular disease, prognosis and drug response. The integrative analysis of various omics data has become increasingly widespread because each approach has intrinsic caveats. For instance, important information may be missing because of false negatives or may be misleading because of false positives. In addition, by analyzing different types of data in isolation we may miss important information that results from the coordinated activity of biological components at various levels. Some studies indicated that these limitations can be mitigated by integrating two or more omics datasets. Several studies (Goh et al., 2007; Lee et al., 2008; Lu et al., 2008; Hu and Agarwal, 2009; Liu et al., 2009; Park et al., 2009; Schadt, 2009; Jiang et al., 2010; Suthram et al., 2010) reported on the role of a single omic or phenotypic measure to represent disease-disease associations (such as shared pathways or gene ontology). But, one needs to study diverse sources of evidence including miRNA-based relationships, shared environmental factors, ontology, SNPs, CNVs and phenotypic manifestations for better understanding.

Since, diseases may share many different types of associations with varying levels of risk for disease comorbidities, a singular view of associations between diseases is not enough to predict comorbidities. As more and more ontology, phenotype, omics and environmental data sets become publicly available, it is beneficial to improve our understanding of human diseases and diseases comorbidities based on these new system-level biological data. Combination of multiple types of omics, phenotype and ontology data identifies integrative biomarkers for the stratification of patients with clinical outcome. Further, behavioral and environmental aspects should also be considered in order to realize disease-disease associations. Therefore, it is clear that method and tool for stratifying patients and prediction of disease comorbidities in order to reliably predict prognosis or success of treatments are of critical importance in the field of medicine. We propose a computational framework that integrates all available, heterogeneous and relevant data including miRNA-target interactions, miRNA-disease association, phenotype similarities of diseases, GO (gene ontology), SNPs, CNVs and known disease-environmental associations to capture the complex relationships between phenotypes, genotypes and clinical comordibidity. Therefore, the underlying goal of this chapter is to integrate diverse sets of omics, environmental and phenotypic data, and to develop the comprehensive models of interaction between the disease associated factors for the prediction of the patient specific disease comorbidity, and to develop comorbidity map.

In the case of a complex or even in an unknown case of diseases, physicians may get assistance to take decision quickly and efficiently by using effective software tool. We developed an R software tool POGO to compute statistically significant associations among diseases, to predict disease comorbidity risk and to develop comorbidity maps, which are useful for the physicians and informative for the patients. To perform the computation of the comorbidity risk, this software uses clinical, gene expression, miRNA, SNPs, CNVs, ontology, phenotypic, and environmental data. The inputs of this software is the initial diagnostic result of the patient. The goal of this software is to construct comorbidity maps that incorporate disease interactions, omics, phenotypic and ontology information, and environmental influences. It is a user-friendly and interactive personalised disease and disease comorbidity prediction software. It provides different comorbidity assessment and stratification; integration of omics information with POGO output data could be used to predict more accurate survival probability of patients. The functions included in POGO offer flexibility for applications, and can be easily integrated into highthroughput analysis pipelines for translation medicine.

## Implementation

**Figure 1:**
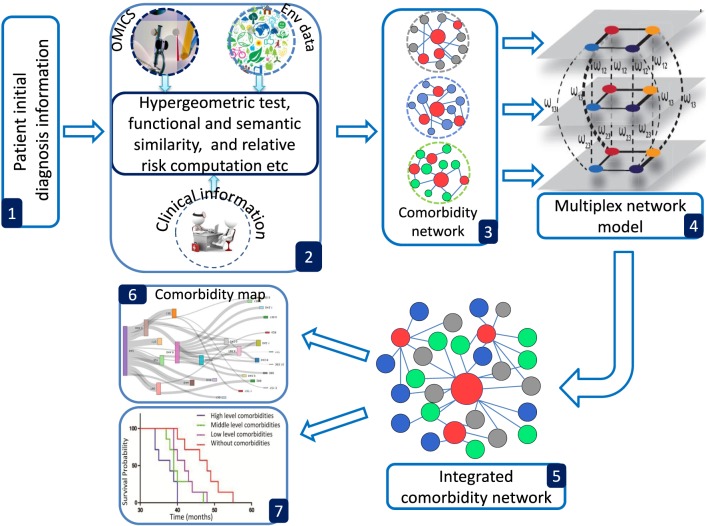
**Overview framework of POGO software**. (1) POGO takes as input preliminary diagnosis data of a patient and check the validation of the input. (2) It preprocesses and updates required databases, performs statistical computation (hypergeometric and semantic similarity tests), and calculates relative risk between diseases. (3) Comorbidity scores and disease network are provided as a result to the user. (4) Multiplex model is applied for data integration to produce integrated comorbidity network as (5). (6, 7) Visualization of the comorbidity map and survival probability of patient considering comorbidity. Env is used to indicate environment.

POGO provides a number of processing options to find comorbidity maps of a patient. R bioconductor annotation data packages “org.Hs.eg.db,” “HPO.db,” and “GO.db” are used for the annotation and mapping between gene symbol, Entrez id, HPO term, OMIM id and GO term (Gentleman et al., 2004). POGO is dependent on “DOSE” and “GOSemSim” bioconductor packages for the mapping with different annotation (Yu et al., 2015). We used the mapping manually constructed by Goh et al. (2007) and Park et al. (2009) to convert OMIM IDs to ICD-9 codes. A set of differential expressed gene symbols/Entrez ids/OMIM id/miRNA ids/HPO terms/GO terms/3 or 5 digit ICD-9-CM code of any disease can be used as input of POGO functions. Flow diagram of POGO software is shown in Figure 1.

### GO–disease association

**Figure 2:**
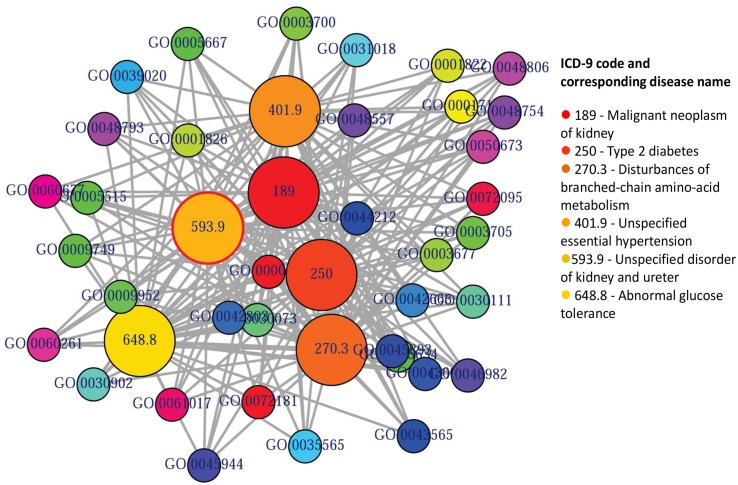
**Output figure and statistics of >comorbidityGO(“189907”, “OMIM”)**. The OMIM disease id of the “Diabetes mellitus, insulin-dependent” is 189907, which is used as input to the comorbidityGO. We show disease comorbidity for the Diabetes mellitus through the GO-disease associations. The size of the nodes represents the degree of associations. ICD-9 codes are used to represent disease categories.

GO enables us to analyse disease association by adopting semantic similarity measures to expand our knowledge of the relationships among different diseases. We downloaded the ontology file and annotations of Homo sapiens from the Gene Ontology database^1^ in April 2014. In total, we collected 171,888 annotations between 13,166 genes and 10,787 GO terms. We developed a function comorbidityGO for the computation of GO based disease comorbidity in an ontology sense. It is a GO-based enrichment analysis function to measure association among diseases and to explore their functional associations from gene sets. We implemented a semantic similarity measurement to quantify the association between gene ontology and their associated diseases. The semantics of GO terms are encoded into a numeric format and the different semantic contributions of the distinct relations are considered. Moreover, hypergeometric test is applied to a gene set to calculate the significance of a GO term, and the significant GO term sets are selected according to their *p*-values. Gene set enrichment analysis are used for predicting the significance of gene–disease and disease–disease associations. comorbidityGO function operates by using either of the following input: GO id, disease OMIM id, a list of gene symbols, Entrez gene ids or ICD-9 code of the patient disease. This function provides disease comorbidity associations and network based on the GO. comorbidityGO requires two parameters: id list and id type. An example and its output is given in Figure 2.


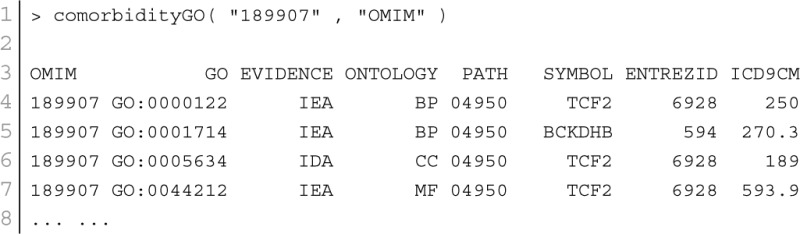


### Phenotype–disease association

**Figure 3:**
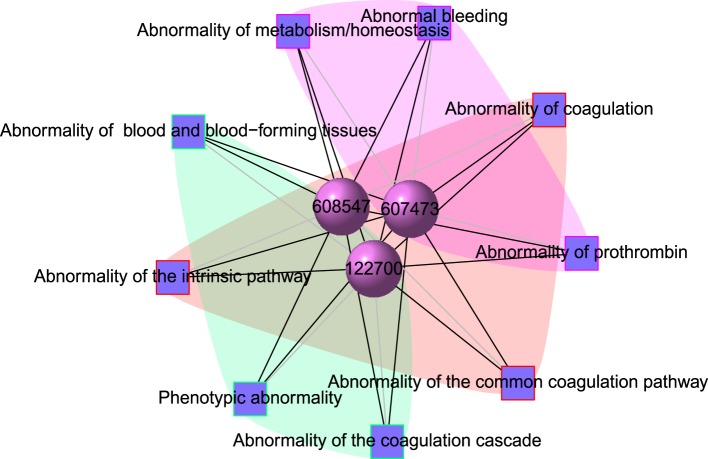
**Output figure and statistics of > comorbidityHPO(“79001”, “Entrez”)**. The Entrez disease id “79001” is used as input to the comorbidityHPO. We show an example of disease comorbidity map for this gene through the phenotype-disease associations. Here the square nodes represent the phenotypes and spheres represent OMIM disease ids.

POGO integrated HPO database that has integrated HPO terms to represent patients phenotypic abnormalities (Robinson et al., 2008). The OMIM (McKusick, 2007) is also incorporated with POGO, and associated to HPO by annotations from http://www.human-phenotype-ontology.org. The associations are generated using the information about the phenotypes of a particular syndrome and the corresponding genes that are known to cause this syndrome when mutated. With the development of omics techniques, the number of uncovered gene-phenotype associations has increased notably over the last few years. In our approach, phenotypes are linked with diseases through associating phenotype-gene with gene-disease bipartite graphs by applying neighborhood-based methods. All the paths from a phenotype to a disease are explored by considering causative genes to assign a weight based on frequency and linked the phenotype to the disease in a new phenotype-disease bipartite graph. Then, we introduced a Bidirectionally-induced Importance Weight prediction method to phenotype-disease bipartite graph in order to approximate the weights of the edges of diseases with phenotypes, by considering link information from both sides of the phenotype-disease bipartite graph. The construction of the phenotype network is based on the phenotypic similarity score among different disease phenotypes. In the phenotype network, the association between any two different disease phenotypes was fixed when their phenotypic similarity score exceeded the significance threshold. For visualization, POGO includes links between disease pairs for which the co-occurrence is notably greater than the random expectation based on phenotype prevalence of the diseases. The function comorbidityHPO of POGO package is able to take input an OMIM id/3 or 5 digit ICD-9-CM code of a disease or a list of gene symbols/Entrez ids and provides comorbidity pattern of diseases based on the phenotype disease associations. comorbidityHPO requires two parameters: id list and id type. An example and its output is given in Figure 3.


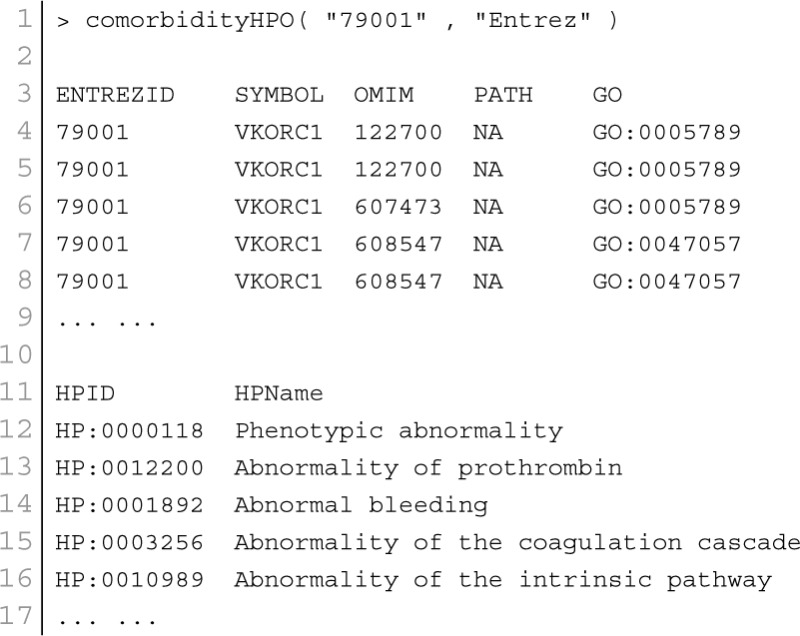


### Disease–SNPs association

**Figure 4:**
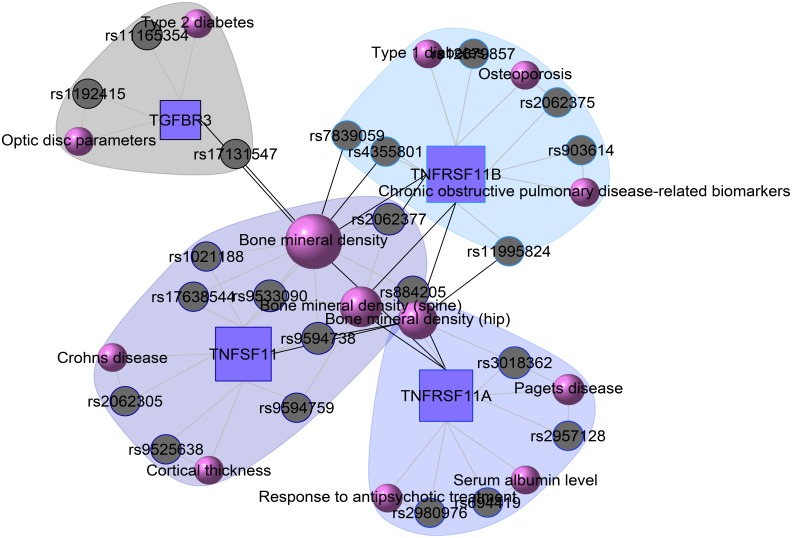
**Output figure and statistics of >comorbiditySNP (c(“TNFSF11”, “TNFRSF11B”, “TNFRSF11A”, “A2M”, “TGFBR3”), “Symbol”)**. We show an example of disease comorbidity through the SNPs-gene-disease associations. Here the square nodes represent the genes symbols, circles represent SNPs ids, and spheres represent diseases names. The size of the nodes represents the degree of associations.

At present there are only a few databases of genetic variations associated with diseases. Despite the needs for analyzing SNP and disease association, most of the existing databases are based only on functional variants at specific locations on the genome, or deal with only a few genes correlated with disease. There is no integrated resource to widely support genes, SNPs, and disease associated information. Therefore, we integrated data from different databases (dbSNP Sherry et al., 2001, HGVbase Fredman et al., 2002, JSNP Hirakawa et al., 2002, GAD Becker et al., 2004 and OMIM McKusick, 2007) and literature Yang et al., 2008 for studying SNPs-diseases associations. We integrated the information to present the interrelationships among SNPs located in genes, genes associated with diseases, and SNPs associated with diseases. It can aid the understanding of the genes which cause diseases and the impact of SNPs on diseases. For associated information among genetic variation and diseases, we built a database, SNP, which is a combined database of genes, genetic variation and diseases for the utilization in POGO. Two diseases are connected if they share at least one SNP that is statistically significant dysregulated to the disease related gene. Our software is designed to capture the relationships between SNPs associated with disease and disease-causing genes. POGO computes disease-disease association by adopting semantic similarity measures and hypergeometric test. Neighborhood based benchmark method is used to identify the comorbidity pattern among diseases (Goh et al., 2007). We built the associated network as a bipartite graph; each common neighbor node is selected based on the Jaccard coefficient method (Goh et al., 2007). comorbiditySNP function of POGO takes as input any of these three options: a list of gene symbols, a list of Entrez gene ids, SNPs ids or an OMIM id. This function provides disease comorbidity associations and network based on the SNPs-gene-disease associations. comorbiditySNP requires two parameters: id list and id type. An example and its output is given in Figure 4.


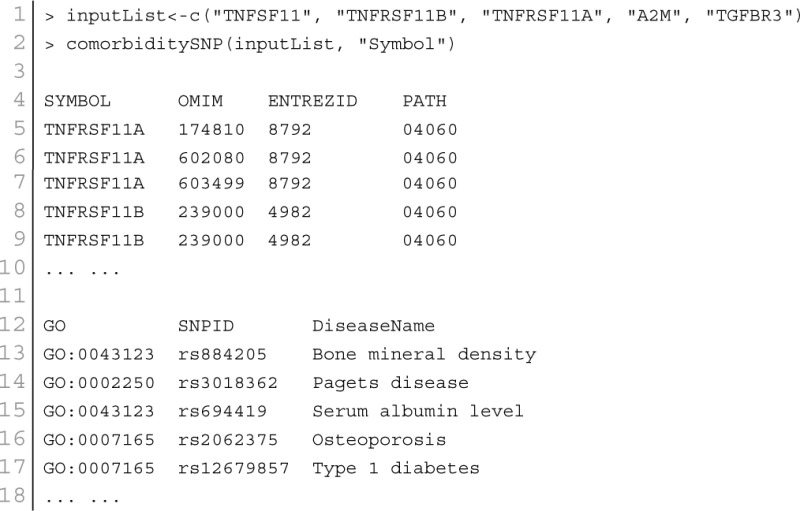


### Disease–environment association

**Figure 5:**
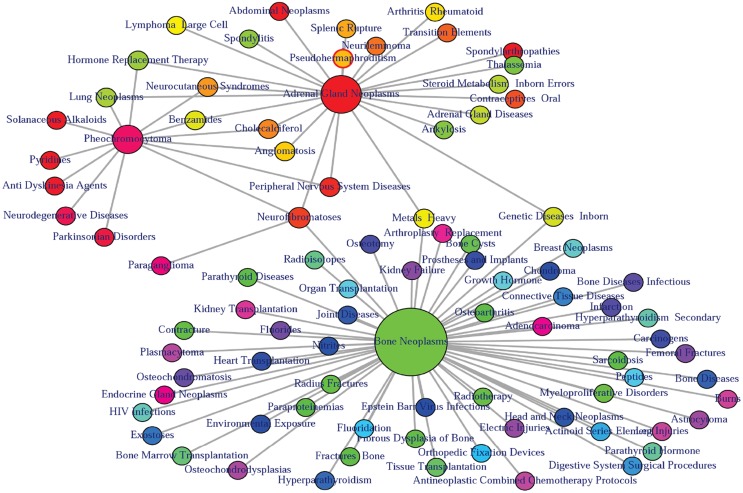
**Output figure and statistics of >comorbidityENV(“SDHB”, “Symbol”)**. The gene symbol “SDHB” is used as input to the comorbidityENV. We show disease comorbidity map for this gene input through the disease-environmental associations. The size of the nodes represents the degree of associations.

The analysis of environment-disease associations is important to investigate the molecular mechanism of a disease. POGO integrated “etiome,” human disease etiological factors database (Liu et al., 2009), and developed a function comorbidityENV to predict the comorbidity risk based on disease environment association (Liu et al., 2009). Integrating genetic, nutritional, behavioral and environmental factors results in the “etiome,” which they defined as the comprehensive compendium of disease etiology (Liu et al., 2009). They used natural language processing to look for annotations in articles, and thus creating associations between diseases and environmental information. “etiome” has been developed with the identified 3342 environment related factors that are associated with 3159 complex diseases (Liu et al., 2009). They also identified 1100 genes associated with 1034 diseases from the genetic association studies database GAD (Becker et al., 2004). GAD has 863 diseases information with both genetic and environmental etiological factors. By using all these information, POGO is able to develop comorbidity map by incorporating relations between the diseases themselves as well as relations to environmental factors. This software identifies the disease–disease associations using the associations among environment and their associated diseases. Hypergeometric test is used for extracting associations among environment and diseases; graph topological structure is used to measure the similarity between diseases (Wang et al., 2007). comorbidityENV function takes as input any of the following options: a list of gene symbols, a list of Entrez gene ids or an OMIM id. This function provides disease comorbidity associations and network based on the gene-environment-disease associations. comorbidityENV requires two parameters: id list and id type. An example and its output is given in Figure 5.


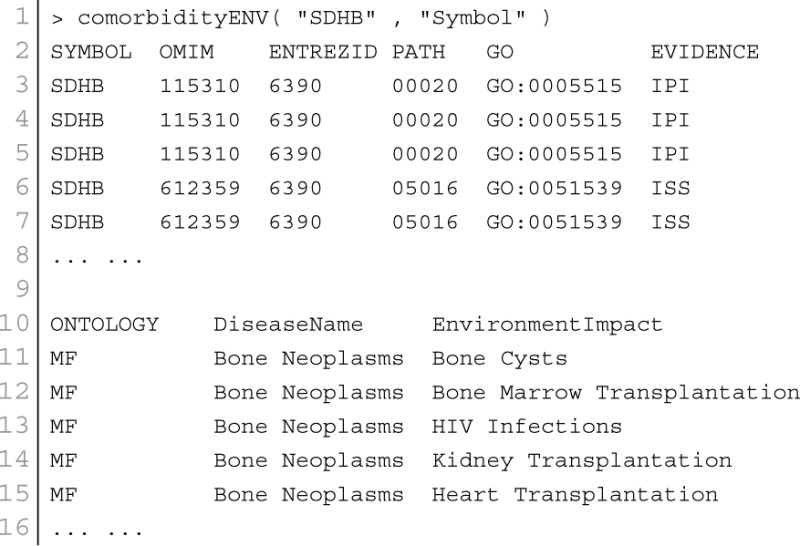


### miRNA–disease association

**Figure 6:**
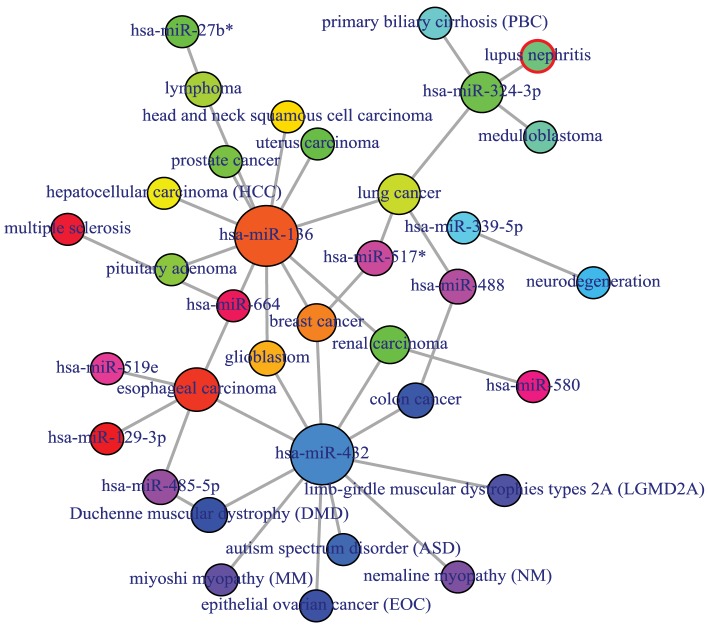
**Output figure and statistics of > comorbiditymiRNA(“TNFRSF11A”, “Symbol”)**. The gene Symbol TNFRSF11A is used as input to the comorbiditymiRNA. We show the comorbidities originated using the miRNA-disease associations information. The size of the nodes represents the degree of associations.

MicroRNA (miRNA) performs its regulatory function through its target genes. Two diseases are connected if they share at least one gene and/or one miRNA that is statistically significant dysregulated (Goh et al., 2007). miRNAs with similar functions tend to be associated with diseases with similar phenotypes, and vice versa (Lu et al., 2008). Based on these hypothesis, we used a framework to identify miRNA-disease associations through the direct identified association from the miRNA-disease association database and indirect association from the combined database of miRNA-target and gene-disease associations. POGO makes use of microRNA-target databases, miR2Disease (Jiang et al., 2009), HMDD (Li et al., 2014), and gene-disease association databases, OMIM (McKusick, 2007), to explore the mRNA and miRNA association between diseases. We filtered out invalid miRNA-disease associations with incorrect disease names or miRNA names. We used National Library of Medicine^2^ to obtain the correct disease names. We used miRBase to get the correct miRNA names (Kozomara and Griffiths-Jones, 2011). For a miRNA-disease pair, firstly, POGO maps the causal genes of the disease. It uses a *p*-value to measure the significance of the association between the miRNA and the disease. OMIM diseases ids are mapped with ICD-9-CM codes based on the literature (Park et al., 2009). Neighborhood based benchmark method is used to identify the comorbidity pattern among diseases. We build the associated network as a bipartite graph; each common neighbor node is selected based on the Jaccard coefficient method (Goh et al., 2007). comorbiditymiRNA function of POGO takes as input any of the following options: a list of gene/miRNA symbols, a list of Entrez gene ids, an ICD-9 code, an GO id or an OMIM id. This function provides disease comorbidity associations and network based on the disease-miRNA associations. comorbiditymiRNA requires two parameters: id list and id type. An example and its output is given in Figure 6.


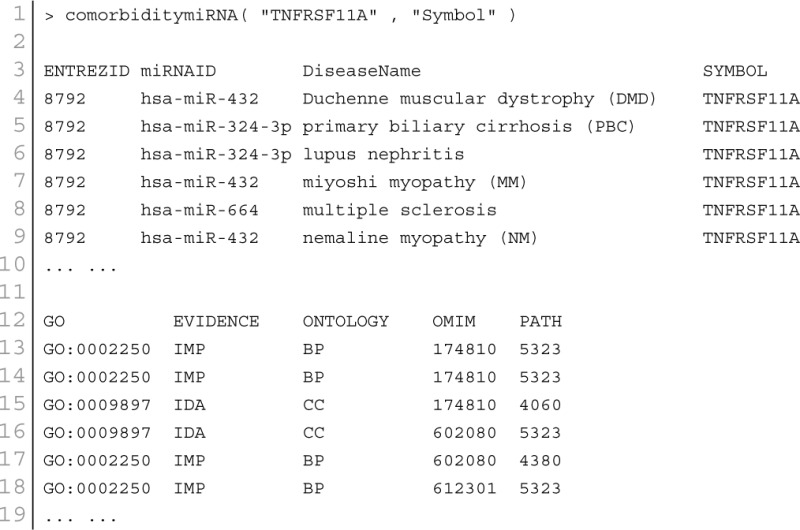


### CNV–disease association

**Figure 7:**
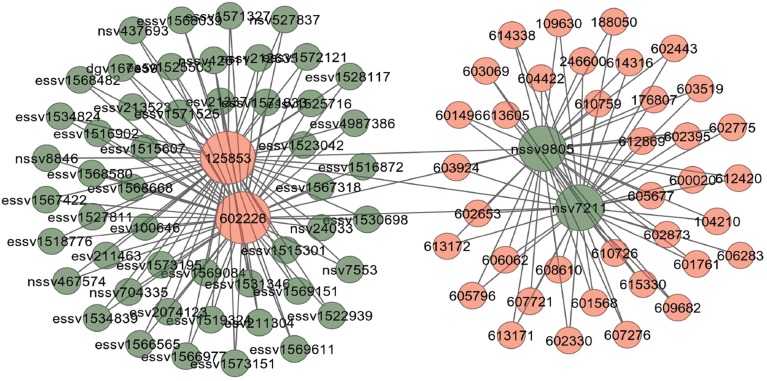
**Output figure and statistics of > comorbidityCNV(“602228”, “OMIM”)**. The OMIM disease id of the “Type 2 Diabetes mellitus” is 602228, which is used as input to the comorbidityCNV. We show disease comorbidity for the “Type 2 Diabetes mellitus” through the CNVs-disease associations. Here the light red color nodes represent the OMIM disease ids and light green color nodes represent the CNVs ids. The size of the nodes represents the degree of associations.

Copy number variants are hypothesized to cause diseases through several mechanisms. Sometimes, the combination of two or more copy number variants can produce a complex disease. Additionally, complex diseases might occur when copy number variants are combined with other genetic and environmental factors (McCarroll and Altshuler, 2007). Diseases might be caused by copy number variants due to both additional copies of sequence (duplications) and losses of genetic material (deletions). We used Database Genomic Variants (DGV^3^) database and developed a function comorbidityCNV to predict the comorbidity risk based on CNVs-disease association (MacDonald et al., 2014). POGO makes use of DGV and OMIM (McKusick, 2007) to explore the genetic association between diseases. Two diseases are connected if they share similar copy number variations. OMIM diseases ids are mapped with ICD-9-CM codes based on the literature (Park et al., 2009). Neighborhood based benchmark method is used to identify the comorbidity pattern among diseases (Goh et al., 2007). We build the associated network as a bipartite graph; each common neighbor node is selected based on the Jaccard coefficient method (Goh et al., 2007). comorbidityCNV function of POGO takes as input any of the following options: a list of gene symbols, a list of Entrez gene ids or an OMIM id. This function provides disease comorbidity associations and network based on the disease-CNV associations. comorbidityCNV requires two parameters: id list and id type. An example and its output is given in Figure 7.


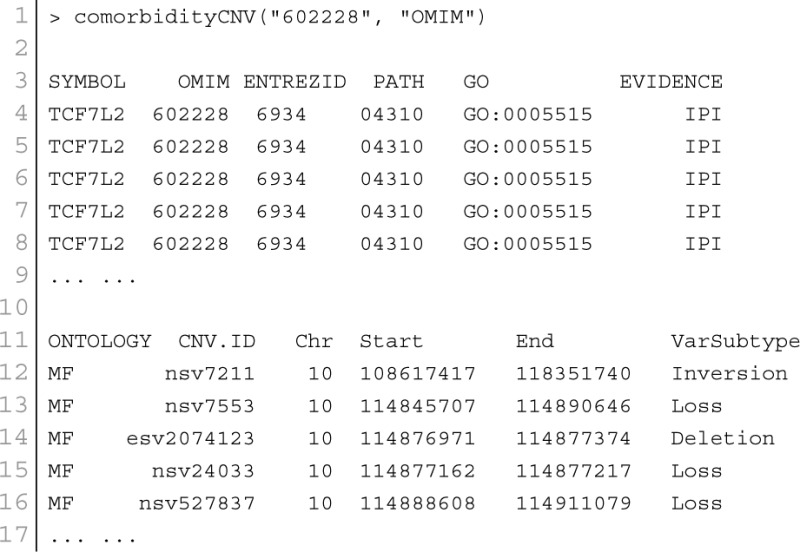


## Integrated comorbidity prediction using multiplex

**Figure 8:**
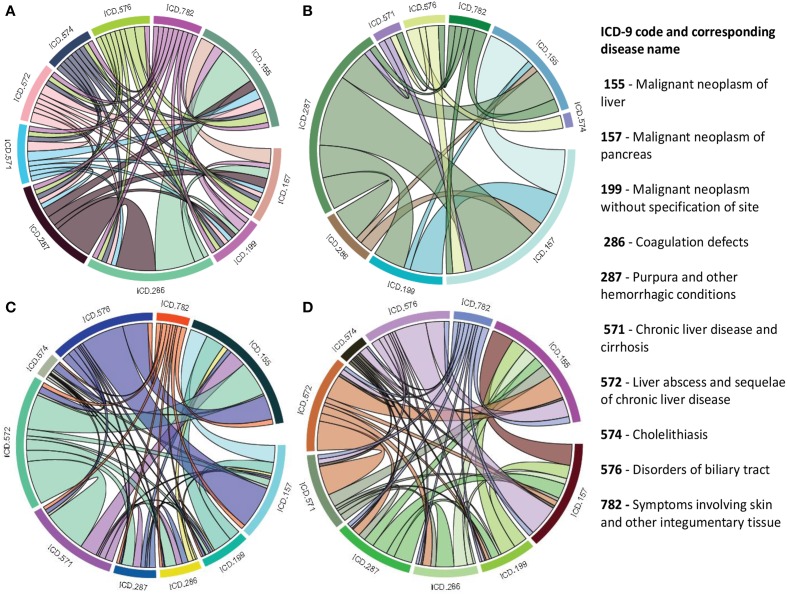
**Disease comorbidities network are constructed by applying the multiplex network model**. Each disease is denoted by the ICD-9-CM code. **(A)** is a comorbidity association network based on the gene disease association data. **(B)** is a comorbidity association network based on the pathway disease association data. **(C)** is a comorbidity association network based on the clinical information. **(D)** is a comorbidity association network based on the integrated multiplex network output of the input of **(A–C)** as layers of the model.

As a single source of genomic data is prone to bias, incompleteness and noise, integration of different genomic data sources is designed to accomplish reliable disease comorbidities prediction. Systematic integration and comparison of multiple layers of information is required to provide deeper insights into biological systems. We incorporated a multiplex network model into POGO to integrate multiple omics, environmental and phenotypic information. To leverage the potential of multi-omics studies, exploratory data analysis methods that provide systematic integration and comparison of multiple layers of omics information are required. We applied our multiplex method of integrating different types of data by modeling similarities between diseases in a multiplex network. The multiplex network allows us to model diseases by representing each data type as a layer in the multiplex. Importantly, this allows us to capture the interactions between the various types of data, such as the interdependence of mRNA expression and signaling pathways with clinical information of the disease comorbidities. We developed a function comorbidityMultiplex to predict the integrated comorbidity risk. comorbidityMultiplex function takes as input any number of layers information. This function provides integrated comorbidity associations and network. As an example of integrating with this function we considered three different types of data for three layers of our multiplex network: mRNA-disease, pathway-disease and clinical association information. An example and its output is given in Figure 8.


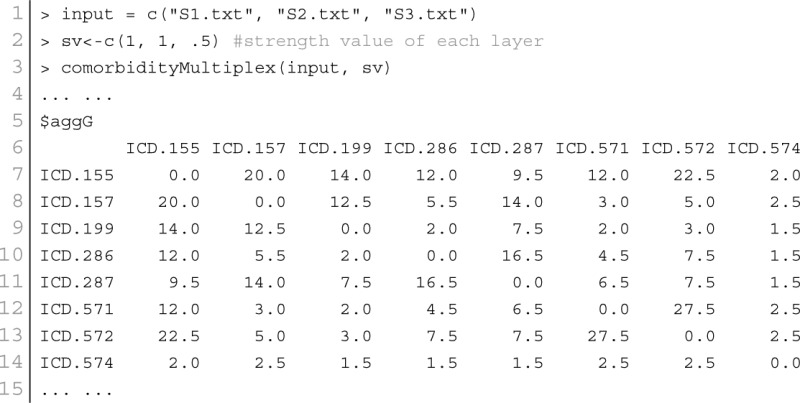


In this example, we considered association information of 10 diseases, which are the output of other functions of POGO. The ICD-9 code of the 10 diseases are 155, 157, 199, 286, 287, 571, 572, 574, 576, and 782. POGO identified disease-disease comorbidity associations network based on the gene-disease association and pathway-disease association, which are shown in Figures 8A,B respectively. It is notable that there is no shared pathway for the disease 572 with the 9 other diseases. The comorbidity network based on the clinical information is shown in Figure 8C. We used all these three association networks for the input of our multiplex network (see Supplementary Tables S1–S3). In this case, the multiplex network is comprised of three layers, each with 10 nodes. In each layer, each node has a weighted undirected edge connecting it to every other node in the same layer. In addition, each disease is connected to itself in every other layer by the strength of interaction between the data types. So the multiplex network created using POGO is formed of three layers using the mRNA, pathway and clinical data. Each layer provided information on the same diseases. This result is a 30 × 30 multiplex matrix, since a multiplex matrix is formed of *n* × *h* rows and columns where *n* is the number of patients and *h* is the number of layers. Our software POGO can find the disease comorbidities by integrating all the descriptive layers, taking into account the properties of the multiplex. All these three categories association data are used as input of our multiplex network and predicted the integrated disease comorbidities network as shown in the Figure 8D.

### Comorbidity mapping

**Figure 9:**
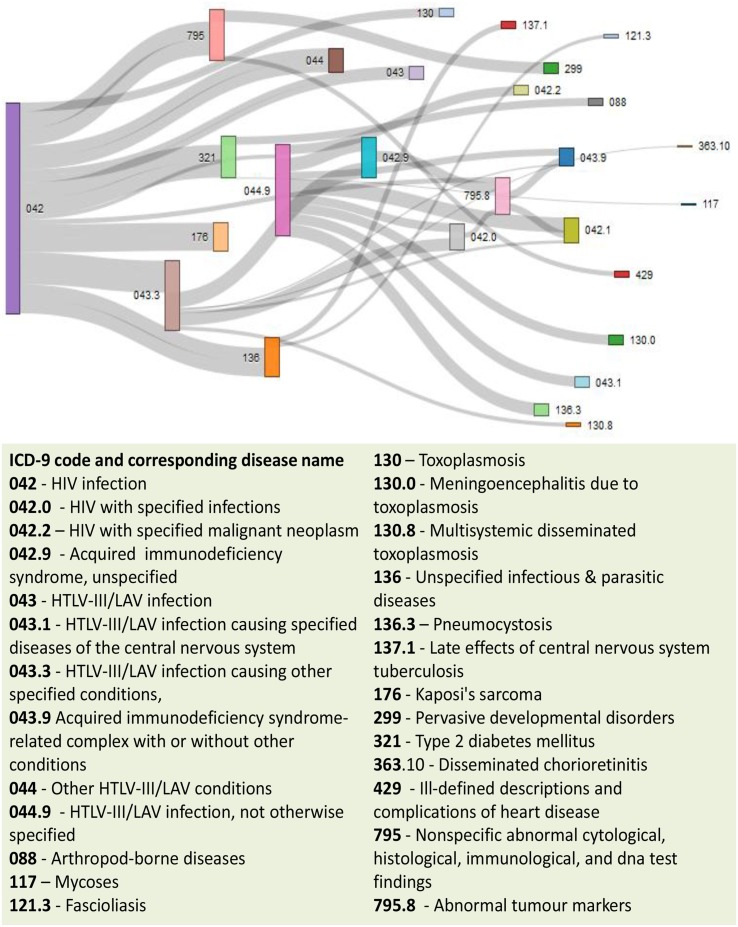
**Output figure and statistics of >comorbidityMap(“042”, “ICD9”)**. The icd-9-CM code of the HIV is 042, which is used as input to the comorbidityMap. We show disease comorbidity for the HIV infection (042) with other diseases, whose ICD-9-CM codes are 042.0 (with specified infections), 042.1 (causing other specified infections), 042.2 (with specified malignant neoplasms), 042.9 (acquired immunodeficiency syndrome, unspecified), 043 (HTLV-III/LAV infection), 043.1 (HTLV-III/LAV infection causing specified diseases of the central nervous system), 043.3 (HTLV-III/LAV infection causing other specified conditions), 043.9 (acquired immunodeficiency syndrome-related complex with or without other conditions), 044 (Other HTLV-III/LAV conditions), 044.9 (HTLV-III/LAV infection, not otherwise specified), 088 (arthropod-borne diseases), 117 (mycoses), 121.3 (fascioliasis), 130 (toxoplasmosis), 130.0 (meningoencephalitis due to toxoplasmosis), 130.8 (multisystemic disseminated toxoplasmosis), 136 (unspecified infectious and parasitic diseases), 136.3 (pneumocystosis), 137.1 (late effects of central nervous system tuberculosis), 176 (Kaposi's sarcoma), 299 (pervasive developmental disorders), 321 (type 2 diabetes mellitus), 363.10 (disseminated chorioretinitis), 429 (ill-defined descriptions and complications of heart disease), 795 (nonspecific abnormal cytological, histological, immunological, and dna test findings), and 795.8 (abnormal tumor markers). POGO uses color rectangle to classify different disease codes and the size of the rectangle is used to represent the severity of that disease.

Patient medical records contain important clarification regarding the co-occurrences of diseases affecting the same patient. Two diseases are connected if they are co-expressed in a significant number of patients in a population (Hidalgo et al., 2009). To estimate the correlation starting from disease co-occurrence, we need to quantify the strength of the comorbidity risk. We used two comorbidity measures to quantify the strength of comorbidity associations between two diseases: (i) the Relative Risk (fraction between the number of patients diagnosed with both diseases and random expectation based on disease prevalence) as the quantified measures of comorbidity tendency of two disease pairs; and (ii) ϕ-correlation (Pearsons correlation for binary variables) to measure the robustness of the comorbidity association (Moni and Lio, 2014). We used the relative risk *RR*_*ij*_ and ϕ-correlation ϕ_*ij*_ of observing a pair of diseases *i* and *j* affecting the same patient. The *RR*_*ij*_ allows us to quantify the co-occurrence of disease pairs compared with the random expectation. When two diseases co-occur more frequently than expected by chance, we will get *RR*_*ij*_ > 1 and ϕ_*ij*_ > 0. The two comorbidity measures are not completely independent of each other. We included links between disease pairs for which the co-occurrence is notably greater than the random expectation based on population prevalence of the diseases. Clinical information is from the http://www.icd9data.com in the ICD-9-CM format and collected from Hidalgo et al. (2009). The function comorbidityMap of POGO package is able to take input an OMIM id/3 or 5 digit ICD-9-CM code of a disease or a list of gene symbols/Entrez ids and provides comorbidity map of the patient based on the relative risk and ϕ-correlation. comorbidityMap requires two parameters: id list and id type. An example and its output is given in Figure 9.


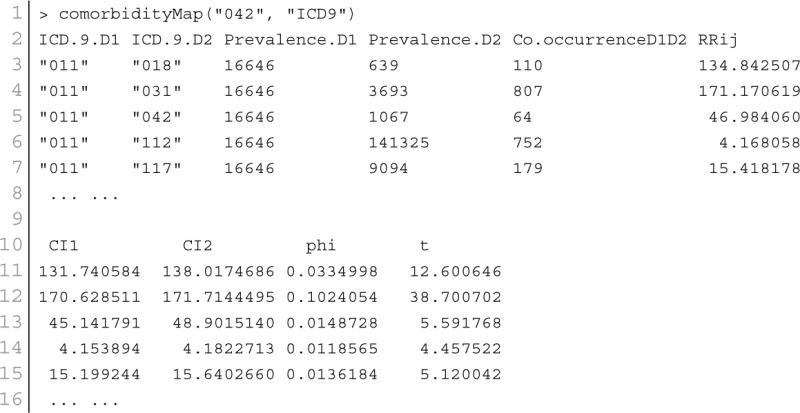


## Methods

Diseases are connected when they share at least one significant dysregulated gene/miRNA/SNP/CNV/GO/phenotype or environmental factor. Let a specific set of associated diseases *D* and a set of significant biomarker genes *G*, gene-disease associations attempt to find whether gene *g* ∈ *G* is associated with disease *d* ∈ *D*. If *G*_*i*_ and *G*_*j*_ are the sets of significant up and down dysregulated genes associated with diseases *i* and *j* respectively then the number of shared dysregulated genes (*n*^*g*^_*ij*_) associated with both diseases *i* and *j* is as follows:

(1) $$n_{ij}^{g}=N(G_{i}\cap G_{j})$$

We calculated the similarity between a pair of diseases based on the number of entities (gene, SNP, CNV, miRNA, HPO or environmental factor) that shared between them. For an instance, in case of gene-disease association, we generated a list of genes known to be associated with each disease, and the disease similarity (association) was calculated based on how many genes are shared between a pair of diseases. The similarity is defined as

(2) $$Sim(i,j)=\frac{N(G_{i}\cap G_{j})}{\sqrt{N(G_{i})}\;*\;\sqrt{N(G_{j})}},$$

where *N*(*G*_*i*_) and *N*(*G*_*j*_) are the number of genes linked to disease *i* and *j* respectively, and *N*(*G*_*i*_ ∩ *G*_*j*_) is the number of genes associated to both disease *i* and *j*. SNP-sharing, CNV-sharing, miRNA-sharing, HPO-sharing and environmental factors were also generated with the same approach used for gene-sharing.

Hypergeometric test is implemented for enrichment analysis (Subramanian et al., 2005). It is used to assess whether the number of selected genes or ontology associated with disease is larger than expected. To determine whether any disease annotate a specified list of genes at frequency greater than what would be expected by chance, POGO calculates a *p*-value using the hypergeometric distribution. Significance of the enrichment analysis is assessed by the hypergeometric test and the *p*-value is adjusted by false discovery rate (FDR). The hypergeometric *p*-value is calculated using the following formula:

(3) $$p-value=1-\sum_{i\;=\;0}^{k\;-\;1}\frac{\left(\begin{array}{c} M\\ i \end{array}\right)\left(\begin{array}{c} N-M\\ n-i \end{array}\right)}{\left(\begin{array}{c} N\\ n \end{array}\right)}$$

where *N* is the total number of reference genes, *M* is the number of genes that are associated to the disease of interest, *n* is the size of the list of genes of interest and *k* is the number of genes within that list which are associated to the disease. In case of GO term the *p*-value reports the likelihood of finding *n* genes annotated with a particular GO term in the set of interest by chance alone, given the number of genes annotated with that GO terms in the reference set. A biological process, molecular function or cellular location which are represented by a GO term is called enriched if the *p*-value is less than 0.05.

The co-occurrence indicates the number of common miRNAs/genes/ontology/SNPs/CNVs between two diseases. We applied the Jaccard index or Jaccard similarity coefficient, which is known as a standard method for comparing the similarity between two sets of entities. Each common neighbor is calculated based on the Jaccard Index method to calculate the strength of co-occurrence, where association score for a node pair is as:

(4) $$Ass_{i,j}=\frac{N(G_{i}\cap G_{j})}{N(G_{i}\cup G_{j})}$$

We improved the performance of the association scores based on the Adamic and Adar measure (Adamic and Adar, 2003), which weights the impact of neighbor disease nodes inversely with respect to their total number of connections as follows:

(5) $$AssScore(i,j)=\sum_{n\in N(G_{i}\cap G_{j})}\frac{1}{log(degree(n))}$$

This inverse frequency technique is based on the principle that rare relationships are more specific and have more impact on the disease association.

Finally POGO calculates disease-disease interaction score. The score refers to the strength of the interaction between the diseases based on the protein interaction. The interaction score (ϕ_*ij*_) is assigned for each disease pair *i* and *j* as follows:

(6) $$\phi_{ij}=log(n_{ij}^{g}\;*\;N+Z)-\log(NG_{i}\;*\;NG_{j}+Z)$$

Here, *NG*_*i*_ and *NG*_*j*_ are the total number of genes for the disease, *i* and *j*, respectively. *n*^*g*^_*ij*_ is the total number of common genes between the two diseases. *N* is the size of entire proteins involved in the disease protein network. *Z* is a constant (*Z* = 1) introduced to avoid out-of bound errors, if *NG*_*i*_ = *NG*_*j*_ = *n*^*g*^_*ij*_ = 0. The expected result of ϕ_*ij*_ is positive, when the disease pair is over-represented and negative, when the disease pair is under-represented. Co-occurrence also indicates the number of shared patients. So, we used weighting scheme to avoid the bias based on disease prevalence. The mutual information weight *W*(*d*_*i*_, *d*_*j*_) between two diseases *d*_*i*_ and *d*_*j*_ is defined as

(7) $$W(d_{i},d_{j})=log\left(\frac{p(d_{i},d_{j})}{p(d_{i})\;*\;p(d_{j})}\right)$$

where the numerator is the observed co-occurrence (joint probability) and the denominator is the random expectation of co-occurrence (product of marginal probabilities).

The use of semantic similarity between biological processes to estimate disease association could enhance the identification and characterization of disease association besides identifying novel biological processes involved in the diseases. Graph-based methods using the topology of GO graph structure is used to compute semantic similarity. We adapted the approach for computing the functional similarity of GO terms from Wang et al. (2007, 2010). Semantic values of GO term are measured according to the DAG of corresponding disorders. Semantic similarity for any pair of GO term is calculated based on disease semantic value. Formally, a GO term *a* can be represented as a graph *DAG*_*a*_ = (*a*, *T*_*a*_, *E*_*a*_), where *T*_*a*_ is the set of all GO terms in *DAG*_*a*_, including term *a* itself and all of its ancestor terms in the GO graph, and *E*_*a*_ is the set of corresponding edges that connect the GO terms in *DAG*_*a*_. To encode the semantic of a GO term in a measurable format to enable a quantitative comparison, Wang firstly defined the semantic value of term *a* as the combined contribution of all terms in *DAG*_*a*_ to the semantics of term *a* (Wang et al., 2007). Terms closer to term *a* in *DAG*_*a*_ contribute more to its semantics (Wang et al., 2010). Thus, the contribution of a GO term *t* in *DAG*_*a*_ is defined to the semantics of GO term *a* as the *S* value of the term *t* related to term *a*, *S*_*a*_(*t*), which can be calculated as:

(8) $$S_{a}(t)=\{\begin{array}{l} S_{a}(a)=1\;if\;t=a\\ S_{a}(t)=max\{w_{e}\;*\;S_{a}(t^{\prime })|t^{\prime }\in childrenof(t)\}if\;t\neq a \end{array}$$

where *w*_*e*_ is the semantic contribution factor for edge *e* (*e* ∈ *E*_*a*_) linking term *t* with its child term *t*′. It is assigned between 0 and 1 according to the types of associations. Term *a* contributes to its own is defined as one. Then the semantic value of GO term *a*, *SV*(*a*) and the semantic value of GO term *b*, *SV*(*b*) are calculated as:

(9) $$SV(a)=\sum_{t\in T_{a}}S_{a}(t),\;SV(b)=\sum_{t\in T_{b}}S_{b}(t)$$

Thus, for the given two GO terms *a* and *b*, the semantic similarity between these two terms is defined as:

(10) $$S_{sim}(a,b)=\sum_{t\in T_{a}\cap T_{b}}\frac{S_{a}(t)+S_{b}(t)}{SV(a)+SV(b)}$$

where *S*_*a*_ (*t*) is the semantic value of term *t* related to GO term *a* and *S*_*b*_(*t*) is the semantic value of GO term *t* associated to GO term *b*. The semantic similarity between two sets of GO terms *A* and *B* is calculated as

(11) $$Sim(A,B)=\frac{1}{\left|A|+|B\right|}\left(\sum_{a\in A}Sim(a,B)+\sum_{b\in B}Sim(b,A)\right)$$

where |*A*| and |*B*| represent the numbers of terms in sets *A* and *B* respectively.

To obtain more insight into the shared risk factors mechanism of associated human genetic diseases, mapping was implemented from disease phenotype to gene based on the disease-gene association. With the integration of huge numbers and diverse set of experimental data, prediction of gene-phenotype interactions has emerged as a very productive subfield with great importance for the understanding of human disease. Given a specific set of human phenotype *D*, a set of human genes *G* and evidence *E*, these approach attempt to find whether gene *g* ∈ *G* is associated with phenotype *d* ∈ *D*. It is notable that *E* could be gene-disease associations obtained through genetic studies. To quantitatively explore the phenotypic similarity between different phenotype records *P*_*i*_ and *P*_*j*_, according to Zhang et al. (2010) we defined the association measure as cosine of the angle between their corresponding phenotype feature vectors using the following formula:

(12) $$Sim(P_{i},P_{j})=\frac{\sum_{k\;=\;1}^{N}w_{k,i}*\;w_{k,j}}{\sqrt{\sum_{k\;=\;1}^{N}{\left(w_{k,i}\right)}^{2}*}\;\sqrt{\sum_{k\;=\;1}^{N}{\left(w_{k,j}\right)}^{2}}}$$

where *N* is the total mapping concepts, *w*_*k*, *i*_ and *w*_*k*, *j*_ were the *k*-th term, weight in phenotype record *P*_*i*_ and *P*_*j*_, respectively.

For each of the phenotype clusters, mapping was implemented from disease phenotypes to their associated disease genes based on the disease-gene association list in the GAD and OMIM databases. Therefore, we can get the corresponding gene subsets mapped to different phenotype clusters. OMIM disease ids were mapped to the hierarchy of HPO to retrieve the matched HPO terms. Then, a new HPO similarity is calculated for each pair of phenotypes by Jaccard similarity Index

(13) $$Sim_{HPO}=\frac{\left|P1\cap P2\right|}{\left|P1\cup P2\right|}$$

where *P*1 and *P*2 are the set of the matched HPO terms of the two phenotypes, respectively.

The way to assign terms to objects is to add annotations. In our case, the entities represent genes and terms corresponding to phenotypes (HPO terms) or biological processes (GO terms). The specificity of the terms associated with genes allows us to calculate the most significant relationships between them, which use to be related to its proximity to the root.

Each disease is generally mapped to multiple phenotypic features. In order to compute associations between two diseases, *d*1 and *d*2, we adapt a method previously developed for estimating protein similarity with GO (Pesquita et al., 2008), where each feature of *d*1 is matched with the most similar feature of *d*2 and the average is taken over all such pairs of features:

(14) $$sim(d1\to d2)=avg\left[\sum_{s\in d1}\underset{t\in d2}{\max}sim(s,t)\right]$$

Equation (14) is not symmetric with respect to *d*1 and *d*2, the final similarity metric is defined as the mean of Equation (14) taken in both orientations:

(15) $$sim(d1,d2)=\frac{1}{2}\;*\;sim(d1\to d2)+\frac{1}{2}\;*\;sim(d2\to d1)$$

This metric is used to indicate the similarity between two disorders, each of which is mapped to multiple HPO terms.

### Multiplex network model for data integration

We developed multiplex network model to integrate diverse set of omics and clinical data to predict disease comorbidities. It is a special type of multilayered network which is called the multiplex network, in which the same nodes are present in all layers, i.e., *V*_1_ = *V*_2_ = …… = *V*_*M*_ = *V* and where nodes can only have interlayer connections to their counterpart nodes, i.e., *E*_αβ_ = (*v*, *v*); *v* ∈ *V* for all α, β ∈ 1, …, *M*, α ≠ β (Boccaletti et al., 2014).

**Figure 10:**
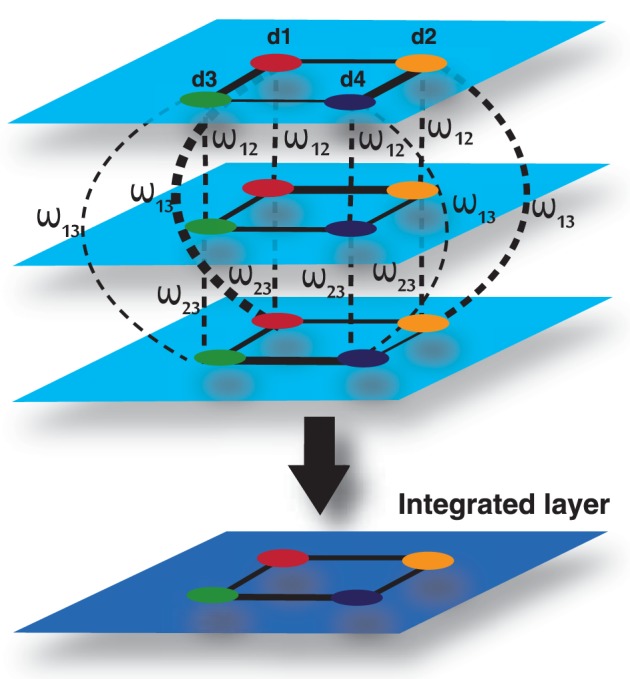
**Multiplex formed by three input layers, each representing a data type, and four nodes, each representing a disease**. The 4th layer is an output layer, which is an integrated layer of the 3 input layers.

Let's consider that we have a set of associated diseases. Each pair of diseases has different types of associated data describing them in some way. In each data type, diseases have some level of association to each other and each data type has a level of dependency or interaction. Each layer in the multiplex represents a particular type of data with each node representing a disease in each layer of the multiplex. The edges between nodes in each layer represent a measure of association between diseases in corresponding to the level of similarity between diseases for the particular data type which the layer represents. The strength of interaction between each data type can be modeled by a weight connecting each layer in the multiplex. Figure 10 shows an example with three layers (data types) and four diseases. In this case we can model the association among diseases in a multiplex network that can be represented in a matrix as follows:

(16) $$M=\left(\begin{array}{cccc} A_{1} & \omega_{12}I & \ldots & \omega_{1h}I\\ \omega_{21}I & A_{2} & \ldots & \omega_{2h}I\\ \vdots & \vdots & \ddots & \vdots \\ \omega_{h1}I & \omega_{h2}I & \ldots & A_{h} \end{array}\right),$$

where *h* is the number of layers, *A*_*i*_ is the adjacency matrix of layer *i*, ω_*ij*_ is the interlayer interaction strength from layer *i* to *j* and *I* is the corresponding identity matrix. The strength between layers in the multiplex, ω, represents a measure of dependency or strength of interaction between the layers. The edge weights between nodes represent a measure of similarity between nodes in the same layer, normalized between zero and one. Therefore, it is natural for the values of ω to represent a measure of dependence between zero and one, where zero and one indicate independence and total dependence between the layers respectively. In our case the strength of interaction is undirected and symmetric, i.e., ω_*i*, *j*_ = ω_*j*, *i*_.

To compute an overall disease similarity between patients given all sets of data, we can find the disease similarity by aggregating the descriptive layers in some way, taking into account the properties of the multiplex. Estrada and Gómez-Gardeñes (2014) defined the aggregate network, $\hat{G}$, of a multiplex network as follows. Let *G*_1_ = (*V*_1_, *E*_1_), *G*_2_ = (*V*_1_, *E*_2_), …, *G*_*h*_ = (*V*_1_, *E*_*h*_) be the set of layers in the multiplex. Then $\hat{G}$ = ($\hat{V}$, $\hat{E}$) where $\hat{V}$ = *V*_1_ and $\hat{E}$ = ∪^*h*^_*i* = 1_ *E*_*i*_. In other words, the aggregate is defined as the union of all edges across all layers of the multiplex. In the literature, the aggregate of a multiplex is often defined in this way. This method can aggregate layers of a multiplex in which the layers are unweighted graphs. However, it is not sufficient for a weighted graph, particularly a complete weighted graph. In addition, the strengths between layers are not accounted for.

Let's consider that the edge weights between nodes provides a normalized measure of similarity between zero and one. We can define the weight of a path between two nodes in the multiplex to be the product of the edges between each node in each step of the path. Since the weight between nodes is a measure of similarity or information shared between the nodes, it follows that the weight of the path provides a measure of information flowing through the path.

There are a number of ways we can provide a new measure of similarity between two nodes given the properties of the multiplex network. One way would be to take the mean of the direct paths connecting each patient to and from another patient in each and every layer. We defined this mathematically as follows:

(17) $$R_{direct}=\frac{\sum_{i\;=\;1}^{h}\left(M{|}_{p_{i}q_{i}}+\sum_{j\;=\;1,j\;\neq \;i}^{h}M^{2}{|}_{p_{i}q_{j}}\right)}{h^{2}},$$

Where *h* is the number of layers in the multiplex, *M*|_*p*_*i*_*q*_*i*__ is the element in the multiplex matrix representing the weight between node *p* and *q* in layer *i* and *M*^2^|_*p*_*i*_*q*_*j*__ is the element in the square of the multiplex network, representing the weight of the path from node *p* in layer *i* to node *q* in layer *j*. Another way would be to take the maximum or minimum information shared directly between two nodes.

(18) $$R_{direct_{min}}=\overset{h}{\underset{i\;=\;1}{\min}}\left(M{|}_{p_{i}q_{i}}+\sum_{j\;=\;1,j\;\neq \;i}^{h}M^{2}{|}_{p_{i}q_{j}}\right)$$

(19) $$R_{direct_{max}}=\overset{h}{\underset{i\;=\;1}{\max}}\left(M{|}_{p_{i}q_{i}}+\sum_{j\;=\;1,j\;\neq \;i}^{h}M^{2}{|}_{p_{i}q_{j}}\right)$$

In many situations, a pair of nodes in a network does not communicate only through the shortest-path routes connecting both nodes, but also through all possible routes connecting both nodes. The number of these possible routes can be enormous. Moreover, the information can also go back and forth before connecting the pair of nodes. Network communicability, which was introduced by Estrada and Gómez-Gardeñes (2014), attempts to quantify such correlation effects in the communication between nodes in complex networks. Estrada and Gomez-Gardenes defined communicability as a measure that “quantifies the number of possible routes that two nodes have to communicate with each other.” In multiplex networks, the communicability, *C*, between two nodes *p* and *q*, is a weighted sum of all walks from *p* to *q*.

(20) $$C_{pq}=I\;+\;M\;+\;\frac{M^{2}}{2!}\;+\;\ldots =\sum_{k\;=\;0}^{k}\frac{M^{k}}{k!}{|}_{pq}.$$

Hence, the communicability between nodes *p* and *q* is given by:

(21) $$C_{pq}={\left[e^{\left(A_{L}\;+\;V_{LL}\right)}\right]}_{pq}={\left[e^{M}\right]}_{pq},$$

where the *p*, *q*-th entry in the minor, ***C***, defines the communicability broadcasted from node *p* in layer *i* to node *q* in layer *j*. Therefore, the communicability broadcasted and received by the nodes in the multiplex is given by:

(22) $$C=e^{\left(A_{L}\;+\;V_{LL}\right)}=\left(\begin{array}{cccc} C_{11} & C_{12} & \ldots & C_{1h}\\ C_{21} & C_{22} & \ldots & C_{2h}\\ \vdots & \vdots & \ddots & \ddots \\ C_{h1} & C_{h2} & \ldots & C_{hh} \end{array}\right)$$

Since all nodes are present in each layer of the multiplex, we can calculate the integrated communicability from node *p* and *q* in all layers in the multiplex by taking the harmonic mean of the communicability between them in each minor in the matrix ***C***.

(23) $${\hat{C}}_{pq}=\frac{h}{\sum_{i\;=\;1}^{h}\frac{1}{{\left[C_{i,i}\right]}_{pq}}+\sum_{j,k\;=\;1,j\;\neq \;k}^{h}\frac{1}{{\left[C_{jk}\right]}_{pq}}}.$$

Hence, the integrated communicability matrix is formed by:

(24) $$\hat{C}=\left(\begin{array}{cccc} 0 & {\hat{C}}_{12} & \ldots & {\hat{C}}_{1h}\\ {\hat{C}}_{21} & 0 & \ldots & {\hat{C}}_{2h}\\ \vdots & \vdots & \ddots & \ddots \\ {\hat{C}}_{h1} & {\hat{C}}_{h2} & \ldots & 0 \end{array}\right),$$

where $\hat{C}$_*ij*_ represents the interaction of layer *i* with layer *j*. Therefore, this multiplex network model is applicable to integrate omics and clinical information of a number of diseases or patients in an efficient way.

## Evaluation

**Figure 11:**
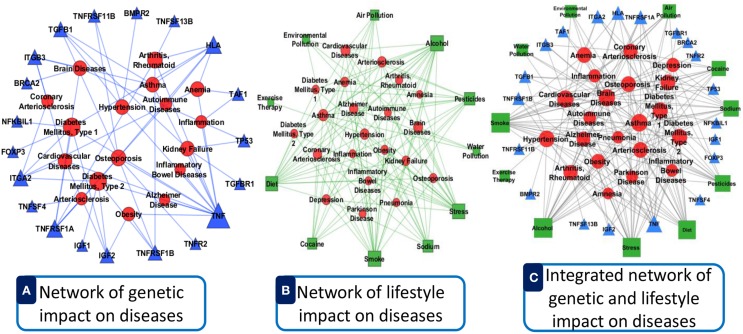
**Comparative representation of dysregulated genes and lifestyle impact on the disease comorbidity**. **(A)** Represents an example of only dysregulated genetic influences on diseases with good lifestyle. **(B)** Shows an example of only bad lifestyle influences on diseases with no genetic variation. **(C)** Represents the combined impacts of lifestyle and dysregulated genes on diseases. Here, the circular red nodes nodes represent diseases name, triangular blue nodes represent genes symbols and square green nodes represent lifestyle factors. The size of the nodes represents the degree of associations.

We incorporated verified data from different data source with our software. Data integration reduces noise associated with each experimental limitation, thus increases sensitivity and specificity to detect true association relationships which results in less number of false positives. By integrating different types of omics and clinical data can produce more reliable predictions with increased sensitivity and specificity for detecting true functional disease comorbidity associations. This can help in finding the hidden connections between complex diseases. Such connections between complex diseases reflect common biological pathways and biological functions that may become manifest in the form of comorbidity. For an example, we show a comparative representation of dysregulated genes and lifestyle impact on the disease comorbidity in Figure 11. Here, panel A (see Figure 11A) represents an example of only dysregulated genetic influences on diseases with good lifestyle. Panel B (see Figure 11B) shows an example of only bad lifestyle influences on diseases with no genetic variation. Panel C (see Figure 11C) represents the combined impacts of lifestyle and dysregulated genes on diseases. Here, we observed that the combined impact of both lifestyle and dysregulated genes influences more and multiway on the diseases and disease comorbidities. It is conceivable that by integrating the data ranging from genotype to multiple levels of phenotypes, more precise and robust stratification of the patients with clinical outcome difference can be achieved.

## Discussion

Development of methods combining omics, ontology and clinical information could assist clinical decision making and represent a large step toward personalized medicine. Proactive and personalized medicine will bring fundamental changes to health care, taking carefully targeted preventative or therapeutic action at the earliest indications of risk or disease. In order to facilitate the necessary changes, better tool is needed for assessing risk and optimizing treatments, which in turn require better understanding of disease interdependencies, genetic influence, and translation into a patient's future. However, most software is designed to make a prediction about a single disease or a class of some specific diseases based on the single omics or clinical information. Phenomizer is a web-based system that produces a ranked list of hereditary diseases, taking a set of clinical features (Köhler et al., 2009). This system only considers the phenotypic annotation to diseases, and semantic similarity metrics to measure phenotypic similarity between query phenotypes and disease phenotypes with the use of the HPO (Robinson and Mundlos, 2010). Another software DGFinder which is used to assess candidate genes in interested chromosome regions for their possibility relating to a given disease (Yuan et al., 2010). It integrated a dataset containing 1045 genes related to 305 diseases. Hidalgo et al. analyzed comorbidity associations using the medical records (Hidalgo et al., 2009). There are some online information retrieval tools, such as AmiGO^4^ and QuickGO^5^, to collect gene annotation data from various databases and manually discover the correlations or similarities of gene products by their biological functions (Binns et al., 2009). FindZebra (Dragusin et al., 2013) is a vertical search engine for rare diseases. This system does not consider the genetic effects on disease or phenotypic effects on genes rather it presents a list of disease documents for a given query of symptoms. CARE uses collaborative filtering methods to predict each patient's disease risks based only on their own medical history and that of similar patient's information (Davis et al., 2010). Recently, a tool KnIT has been developed for the complete medical literature knowledge integration (Spangler et al., 2014). DisGeNET is a coherent tool that analyses and interprets human gene network to disease network (Bauer-Mehren et al., 2010). It is able to display gene-disease association networks as bipartite graphs and provides gene centric and disease centric views of the data.

An R package “comorbidities” is able to categorize ICD-9-CM codes based on published 30 comorbidity indices using Deyo adaptation of Charlson index and the Elixhauser index (Deyo et al., 1992; Elixhauser et al., 1998). Our previous R package comoR that provides relative risk, ϕ-correlation, associated genes and pathway between the comorbidity diseases (Moni and Lio, 2014). It is limited to gene expression and pathway molecular data. To our knowledge, there is no available complete software tool for the prediction of disease comorbidities maps based on the multiple omics, gene ontology, phenotype and environmental influences. So, we developed POGO, another R package that implements different statistical approach for the prediction of disease comorbidity maps by integrating diverse set of data. This software could provide comorbidity mapping among all diseases using ontology, miRNA, SNPs, CNVs, phenotypic and environmental information. This software also incorporated a prediction model that explores the past medical patient history to determine the risk of patients to develop future diseases.

Patient's omics data is becoming important for clinical decision making, including disease risk assessment, disease diagnosis and subtyping, drug therapy and dose selection (Ullman-Cullere and Mathew, 2011). In the near future, physician will have to consider omics implications to patient care throughout their clinical work flow, including electronic prescribing of medications. In the not-so-distant future, as we move in to an era of personalized and preventive medicine, healthy individuals may be tracked by multiple layers of omic and clinical data in an effort to track potential disease progression. Our software tool incorporated an integrated framework to establish the associations between genetic diseases and ontology information, which may help to uncover the molecular mechanisms of genetic diseases. The identified disease patterns from POGO could be useful for further investigations with regards to their diagnostic utility or help in the prediction of novel therapeutic targets. Therefore, POGO could be helpful for the personalized medicine system. They are able to detect many diseases at the earliest detectable phase, weeks, months, and maybe years before symptoms appear. POGO could easily be integrated into pipelines for high-throughput analysis, such as Galaxy, and other gene expression data mining, protein interactions validation, predicting causal relationships among phenotypes and miRNA-regulated network interpretation. The underlying hypothesis behind this line of research is that once we catalog all disease-disease relations through the omics, ontology, phenotypic and environmental influence, we will be able to predict the susceptibility of each individual to future diseases using various molecular biomarkers, ushering us into an era of predictive medicine.

Thus, a combination of genetic, ontology and population-level data and information could be analyzed by this software tool to establish and study novel hypotheses about unknown disease mechanisms and disease comorbidity. Understanding how different diseases relate to each other will not only provide us with a global view of disease associations, but also provide potentially new insights into the etiology, classification, and design of novel therapeutic interventions. This has led to the advent of stratified medicine, which translates advances in basic research by targeting etiological mechanisms underlying diseases. Method and tool for stratifying (classifying) patients in order to reliably predict prognosis or success of treatments are of critical importance in the field of medicine. However, with the identification of the new omics and clinical information, we need to update the integrated databases of the POGO. Using the temporal data explored by the time dimension approach, POGO could be extended to predict the time of expected disease diagnosis in addition to the likelihood of occurrence. The result is a patient stratification could be based on more complete profiles than the primary diagnosis. Therefore, POGO is useful for the stratified medicine.

## Conclusion

Integration of multi-omics, ontology and phenotypic information is important for comorbidity prediction and patient stratification. Therefore, our methodological framework and software for integrating genetic and clinical data could be applicable in clinical decision making for personalized medicine. We expect that this combined approach may increase accuracy and decrease effort for disease comorbidity diagnosis. POGO software tool provides robust approaches to study disease comorbidity mappings by integrating omics, phenotype and ontology information, which can be easily integrated into pipelines for high-throughput and clinical data analysis, and to predict causal inference of a disease. This software tool will help to gain a better understanding of the complex pathogenesis of disease risk phenotypes and the heterogeneity of disease comorbidities. Moreover, the disease comorbidity patterns identified using this software tool could be useful for diagnostic utility or to help in the prediction of novel therapeutic targets. Thus, this software tool could be applicable in personalized medicine and clinical bioinformatics. So our software tool for comorbidity diagnosis and patient stratification could result in effective aids to the health practice. This will not only result in improving health outcomes of the patient, but also in reducing the health care costs.

## Availability and requirements

The software package POGO has been written in the platform independent R programming language. It requires R version 2.16 or newer to run. The software is freely available at www.cl.cam.ac.uk/~mam211/POGO/ and will appear in Comprehensive R Archive Network (CRAN) at (http://cran.r-project.org/).

## Funding

This work is supported by the EU Mission T2D project.

### Conflict of interest statement

The authors declare that the research was conducted in the absence of any commercial or financial relationships that could be construed as a potential conflict of interest.

## Footnotes

^1^http://www.geneontology.org.

^2^http://www.nlm.nih.gov/.

^3^http://dgv.tcag.ca/.

^4^http://www.godatabase.org.

^5^http://www.ebi.ac.uk/ego/.

## Supplementary material

Table S1 **Data of disease-disease associations based on the shared genes**.

Table S2 **Data of disease-disease associations based on the shared pathways**.

Table S3 **Data of disease-disease associations based on the clinical information**.
